# Diagnostic and Prognostic Role of miR-192 in Different Cancers: A Systematic Review and Meta-Analysis

**DOI:** 10.1155/2021/8851035

**Published:** 2021-02-04

**Authors:** Lili Wang, Yuhan Liu, Chen Lyu, Alexander Buchner, Heike Pohla

**Affiliations:** ^1^Laboratory of Tumor Immunology, LIFE Center, LMU Klinikum, University Munich, Germany; ^2^Department of Urology, LMU Klinikum, University Munich, Germany

## Abstract

**Introduction:**

It has been shown that miR-192 is abnormally expressed in a variety of cancer types and participates in different kinds of signaling pathways. The role of miR-192 in the diagnosis and prognosis of cancer has not been verified. This article is aimed at exploring the diagnostic and prognostic value of miR-192 through a systematic review and meta-analysis.

**Methods:**

A systematic search was performed through PubMed, Embase, Web of Science, and Cochrane Library databases up to June 16, 2020. A total of 16 studies were enrolled in the meta-analyses, of which 11 articles were used for diagnostic meta-analysis and 5 articles were used for prognostic meta-analysis. The values of sensitivity and specificity using miR-192 expression as a diagnostic tool were pooled in the diagnostic meta-analysis. The hazard ratios (HRs) of overall survival (OS) with 95 confidence intervals (CIs) were extracted from the studies, and pooled HRs were evaluated in the prognostic meta-analysis. Eleven studies including 667 cancer patients and 514 controls met the eligibility criteria for the diagnostic meta-analysis. Five studies including 166 patients with high miR-192 expression and 236 patients with low miR-192 expression met the eligibility criteria for the prognostic meta-analysis.

**Results:**

The overall diagnostic accuracy was as follows: sensitivity 0.79 (95%CI = 0.75-0.82), specificity 0.74 (95%CI = 0.64-0.82), positive likelihood ratio 3.03 (95%CI = 2.11-4.34), negative likelihood ratio 0.29 (95%CI = 0.23-0.37), diagnostic odds ratio 10.50 (95%CI = 5.89-18.73), and area under the curve ratio (AUC) 0.82 (95%CI = 0.78-0.85). The overall prognostic analysis showed that high expression of miR-192 in patients was associated with positive survival (HR = 0.62, 95%CI : 0.41-0.93, *p* = 0.020).

**Conclusion:**

Our results revealed that miR-192 was a potential biomarker with good sensitivity and specificity in cancers. Moreover, highly expressed miR-192 predicted a good prognosis for patients.

## 1. Introduction

Cancer is threatening human health and shorting human life. Around 1.8 million new cancer cases and 60 thousands of cancer deaths occurred in the United States based on the cancer statistics 2020 [1]. One reason for tumor death is that the tumor is already in its advanced stage as soon as it is discovered. In this case, it is important to find a marker that can detect tumors sensitively. However, a sensitive tumor biomarker is lacking in clinical practice. At present, more and more attention has been focused on microRNAs, which are highly conserved, short noncoding RNAs. MicroRNA binds to the 3′ untranslated region of target mRNA by base pairing matching, resulting in degradation of target mRNA or inhibition of protein translation, which is involved in biological progress such as cell growth, differentiation, proliferation, and apoptosis [2]. Besides, miRNAs have been reported to regulate the key characteristics of cancer, involving self-sufficiency in growth signal, antigrowth signal, evasion from apoptosis, limitless replicative potential, angiogenesis, invasion, and metastasis [3]. Therefore, miRNAs could be promising biomarkers for diagnosis and prognosis [4].

MicroRNA-192 was firstly confirmed by Lim et al. in 2003 [5]. It is reported to be overexpressed in gastric cancer [6], hepatocellular carcinoma [7], and neuroblastoma [8], but downregulated in colorectal cancer [9] and lymphoblastic leukemia [10]. MicroRNA-192 plays a critical role in cell proliferation, migration and invasion [11], apoptosis [12], and epithelial-to-mesenchymal transition [13]. More importantly, miR-192 has been consistently detected in sputum [14], cervical cancer tissue, serum [15], and urine [16], suggesting that miR-192 might be a valuable biomarker for cancer diagnosis and detection. However, no meta-analyses concerning association between miR-192 expression and cancer diagnosis and prognosis have been published. Here, we conducted the diagnostic and prognostic meta-analyses to assess the diagnostic and prognostic value of miR-192.

## 2. Materials and Methods

### 2.1. Literature Search Strategy

A systematic literature search was carried out in PubMed, Embase, Web of Science, and Cochrane Library databases up to June 2020. The first part was to screen articles that explored the diagnostic value of miR-192 in cancers. Both MeSH terms and free-text words were used in the search strategy. The following search keywords were used in combination: “neoplasms” or “tumor” or “cancer”, “diagnosis”, “ROC curve”, “sensitivity and specificity”, and “microRNA-192”. The second part was to screen articles that explored the prognostic value of miR-192 in cancers. The keywords were as follows: “neoplasms” or “tumor” or “cancer”, “survival”, “prognosis”, “recurrence”, and “microRNA-192”.

### 2.2. Inclusion and Exclusion Criteria

For diagnostic meta-analysis, studies were included for further evaluation if they meet the following criteria: (1) any types of cancers concerning miR-192, (2) inclusion of a diagnostic standard, (3) sufficient data (true positive, false positive, false negative, and true negative) for calculating the sensitivity and specificity, (4) studies based on humans, and (5) studies published in English. Exclusion criteria were as follows: (1) non-English articles; (2) other types of articles such as conference abstracts, reviews, meta-analysis, patents, case reports, comments, and letters; and (3) insufficient data for calculating the sensitivity and specificity.

For prognostic meta-analysis, studies were included for further evaluation if they meet the following criteria: (1) any types of cancers concerning miR-192, (2) inclusion of a diagnostic standard, (3) associations between the expression of miR-192 and prognosis of patients being determined, (4) hazard ratios (HRs) and their corresponding 95% confidence intervals (CIs) being evaluated, and (5) English publications. Exclusion criteria were as follows: (1) non-English articles; (2) other types of articles such as conference abstracts, reviews, meta-analysis, patents, case reports, comments, and letters; (3) insufficient data to calculate the HRs and 95% CIs; and (4) the prognostic data based on TCGA dataset.

### 2.3. Data Extraction and Quality Assessment

All studies were independently selected by two investigators (Lili Wang and Yuhan Liu), and uncertain data were reviewed by a third author (Chen Lyu). The following information was collected in diagnostic analysis: first author's name, publication year, nationality, ethnicity, cancer type, sample type, test method, cut-off, case number, sensitivity, and specificity. For the prognostic analysis, the following information was collected: first author's name, publication year, nationality, cancer type, cases of high expression of miR-192, cases of low expression of miR-192, the endpoint of follow-up, and HRs along with their corresponding 95% CIs.

The Quality Assessment of Diagnostic Accuracy Studies 2 (QUADAS 2) tool [17] was used to assess the quality of articles included in the diagnostic meta-analysis. The Newcastle–Ottawa Scale (NOS) [18] was used to assess the articles in the prognostic meta-analysis.

### 2.4. Statistical Analysis

All data analyses were performed using Stata MP 16.0 software (StataCorp, College Station, TX). For the diagnostic meta-analysis, the pooled sensitivity, specificity, diagnostic odds ratio (DOR), positive likelihood ratio (PLR), and negative likelihood ratio (NLR) were generated through bivariate meta-analysis. The summary receiver operator characteristic (SROC) curve and the area under the curve (AUC) were calculated to evaluate the overall diagnostic value of miR-192 in cancers. The heterogeneity test was conducted using the chi-square-based *Q* test and Higgins *I*-squared statistic. That *I*^2^ > 50%, and *p* < 0.10 indicated heterogeneity among studies. Publication bias was evaluated by funnel plots and by Begg's and Deeks' tests. *p* < 0.05 suggests the existence of publication bias in studies. For the prognostic meta-analysis, the pooled HRs and 95% CIs were determined using the *Z*-test, with *p* < 0.05 defined as significant. HR > 1 indicated poor prognosis for patients with miR-192, while HR < 1 meant a protective effect for the prognosis of highly expressed miR-192. The methods for the assessment of heterogeneity and publication bias were the same as those for the diagnostic meta-analysis.

## 3. Results

### 3.1. Study Selection and Characteristics

**Figure 1:**
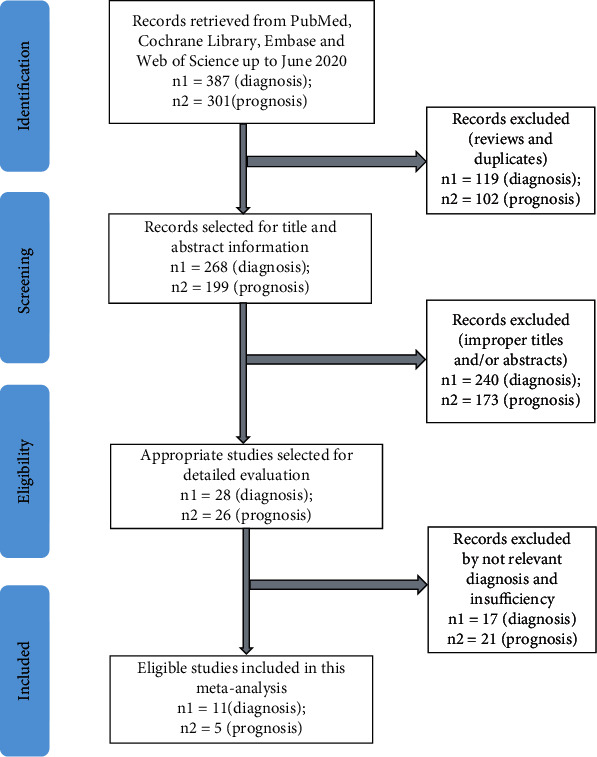
Study selection flowchart.

A total of 387 articles were searched using the search strategy, of which 11 articles [14–16, 19–26] met the inclusion criteria for diagnostic meta-analysis (Figure 1) and included 667 cancer cases and 514 controls. The characteristic details of these articles are summarized in Table 1. The studies involved different types of cancer: non-small-cell lung cancer (*n* = 2, NSCLC), cholangiocarcinoma (*n* = 3, CCA), hepatocellular carcinoma (*n* = 1, HCC), pancreatic ductal adenocarcinoma (*n* = 1, PDAC), pancreatic cancer (*n* = 1, PC), cervical cancer (*n* = 1, CC), bladder cancer (*n* = 1, BC), and acute myeloid leukemia (*n* = 1, AML). The expression of miR-192 was evaluated through qRT-PCR in the tissue (*n* = 3), serum (*n* = 6), and urine (*n* = 2).

For the prognostic meta-analysis, 301 articles were obtained from four databases, and only 5 articles ([23, 27–30]) met the inclusion criteria (Figure 1) and included 166 high-miR-192 cases and 236 low-miR-192 cases. Details concerning the included articles are displayed in Table 2. The studies included several types of cancer: gastric cancer (*n* = 1, GC), colon cancer (*n* = 1, COC), small-cell lung cancer (*n* = 1, SCLC), hepatocellular carcinoma (*n* = 1, HCC), and acute myeloid leukemia (*n* = 1, AML). The expression of miR-192 was assessed through qRT-PCR using plasma (*n* = 1), tissue (*n* = 3), and serum (*n* = 1).

### 3.2. Quality Assessment of Studies

The quality of diagnostic meta-analysis was assessed using the QUADAS-2 tool. All the studies were scored between 4 and 6 points which represented moderate or high quality (Table 1). For the prognostic meta-analysis, the Newcastle–Ottawa Scale (NOS) tool was used to assess the quality of studies according to three elements: selection (0-4 points), comparability (0-2 points), and outcome (0-3 points). All the studies were assessed as moderate or high quality, with scores between 5 and 7 points (Table 3).

### 3.3. The Results of the Diagnostic Meta-Analysis

**Figure 2:**
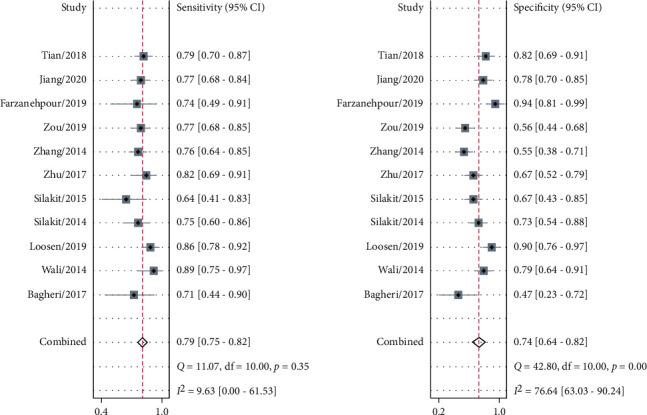
Forest plot of pooled sensitivity and specificity for 11 studies in the diagnostic meta-analysis.

**Figure 3:**
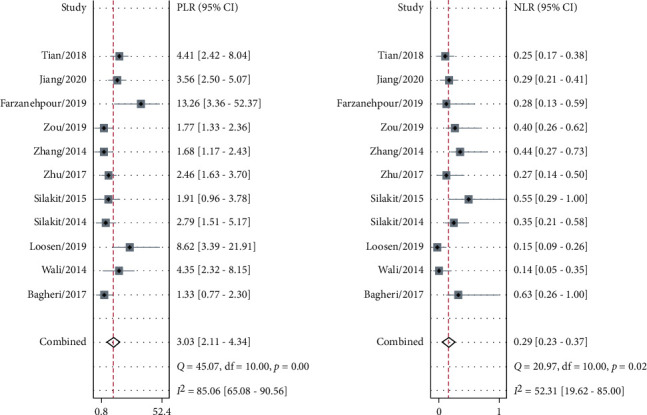
Forest plot of the positive likelihood ratio (PLR) and negative likelihood ratio (NLR) for miR-192 in the diagnostic meta-analysis.

**Figure 4:**
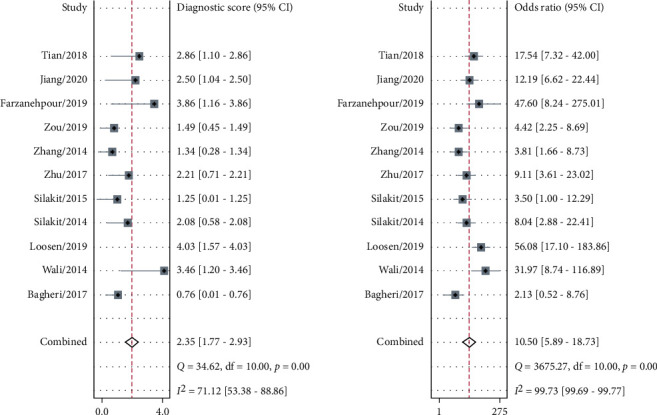
Forest plot of the diagnostic odds ratio for miR-192 in the diagnostic meta-analysis.

**Figure 5:**
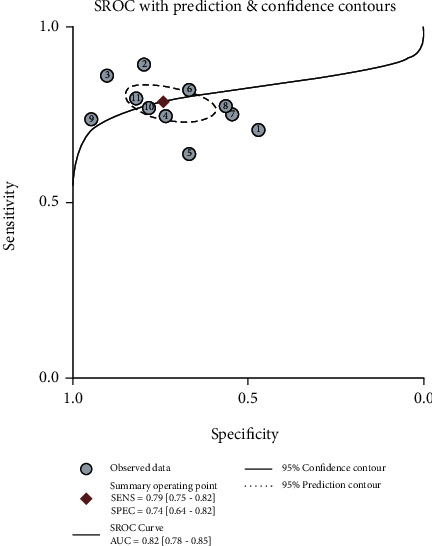
SROC curve for miR-192 in the diagnostic meta-analysis. SENS: pooled sensitivity; SPEC: pooled specificity; AUC: area under the curve.

**Figure 6:**
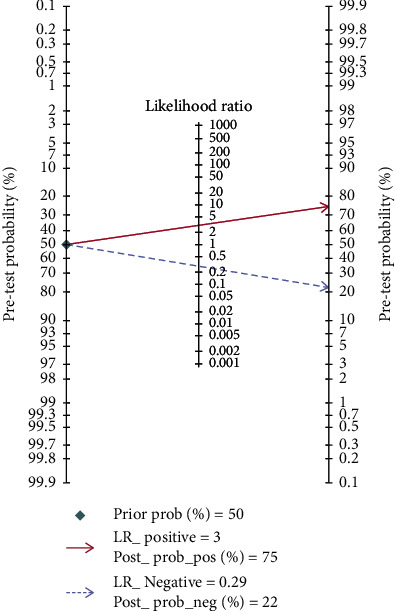
Fagan's nomogram was used to assess the post-test probabilities. LR: likelihood ratio.

The sensitivity and specificity of 11 studies are presented in the forest plots as shown in Figure 2. There was no heterogeneity in the sensitivity (*I*^2^ = 9.63%, 95%CI = 0-61.53%), but significant heterogeneity in the specificity (*I*^2^ = 76.64%, 95%CI = 63.03%-90.24%). Overall, the sensitivity and specificity for the pooled data were 0.79 (95%CI = 0.75-0.82) and 0.74 (95%CI = 0.64-0.82). In addition, the pooled PLR was 3.03 (95%CI = 2.11-4.34), and the NLR was 0.29 (95%CI = 0.23-0.37) as shown in Figure 3. The DOR was 10.50 (95%CI = 5.89-18.73, Figure 4). The SROC curve is shown in Figure 5. The AUC for the miR-192 test method was 0.82 (95%CI = 0.78-0.85), suggesting that miR-192 has a relatively high diagnostic value. Fagan's nomogram was applied for assessing the clinical utility of the index test shown in Figure 6. When miR-192 was tested in patients with a pretest probability of cancer of 50%, the posttest probability of having cancer was improved to 75% by a positive result, while the posttest probability without cancer was dropped to 22% by a negative result. Taken together, miR-192 had a relatively moderate accuracy for identification of cancer patients.

### 3.4. Influence Analysis and Robustness Test

**Figure 7:**
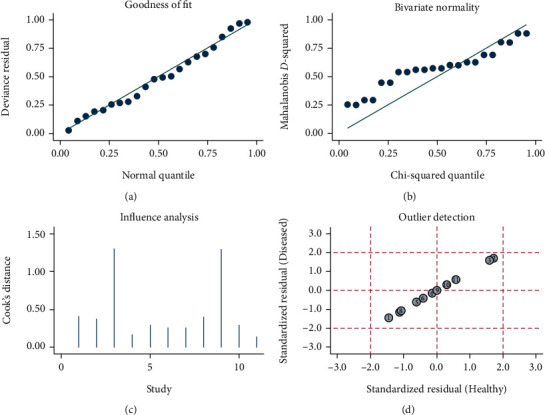
Sensitivity analyses: (a) goodness-of-fit; (b) bivariate normality; (c) influence analysis; (d) outlier detection.

The goodness-of-fit and bivariate normality (Figures 7(a) and 7(b)) analyses suggested that the bivariate model was moderately robust. Influence analysis (Figure 7(c)) and outlier detection (Figure 7(d)) did not identify any outliers.

### 3.5. Publication Bias

**Figure 8:**
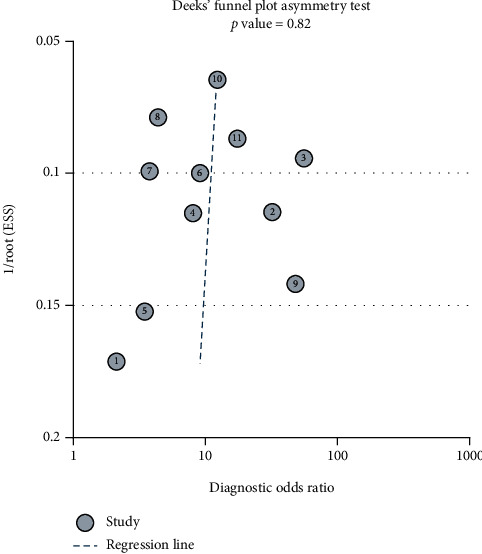
Deeks' funnel plot asymmetry test showed that the *p* value was 0.82 indicating that there was no publication bias.

Deeks' funnel plot asymmetry test suggested that there was no significant publication bias (*p* = 0.82, Figure 8).

### 3.6. Threshold Effect and Heterogeneity

**Figure 9:**
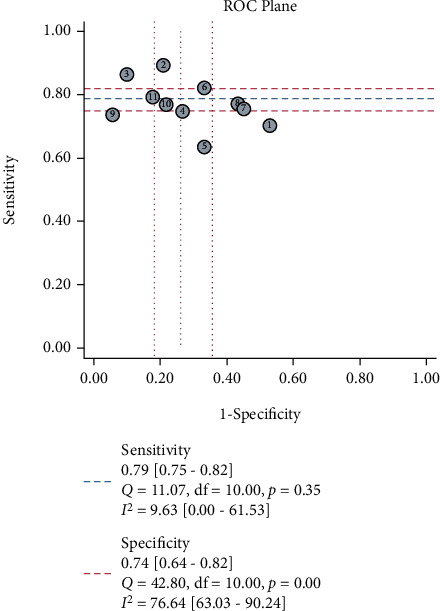
ROC plane showed the results of sensitivity, specificity, *Q* test, and *I*^2^ result.

**Figure 10:**
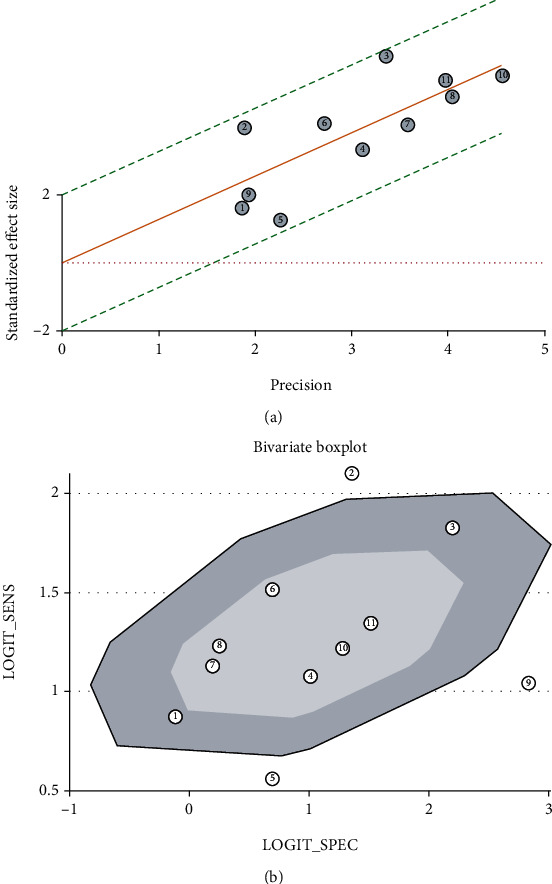
Heterogeneity test: (a) the Galbraith radial plot showed that all the studies were in the 95% CI region suggesting no heterogeneity; (b) there was heterogeneity for three studies beyond the middle region in the bivariate boxplot.

The ROC plane showed the appearance of a nontypical shoulder arm suggesting no threshold effect existing (Figure 9). Spearman's correlation coefficient was –0.374 (*p* = 0.258), also indicating no threshold effect existing. The Galbraith radial plot showed that all the studies were in the 95% CI region suggesting no heterogeneity (Figure 10(a)). The bivariate boxplot showed that most studies were scattered in the middle region except three studies suggesting that there was heterogeneity between studies (Figure 10(b)). Due to only 11 studies included in the diagnostic meta-analysis, it was difficult to perform the subgroup and meta-regression analyses to investigate the sources of heterogeneity.

### 3.7. A Prognostic Meta-Analysis of the Relationship between miR-192 Expression and Prognosis in Cancers

**Figure 11:**
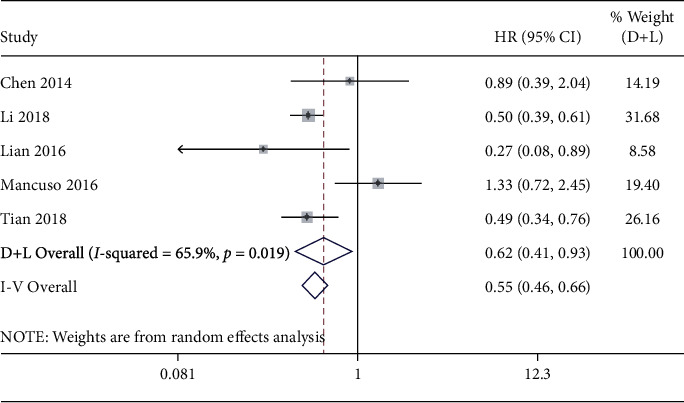
Forest plot of pooled HRs for miR-192 in the prognostic meta-analysis.

**Figure 12:**
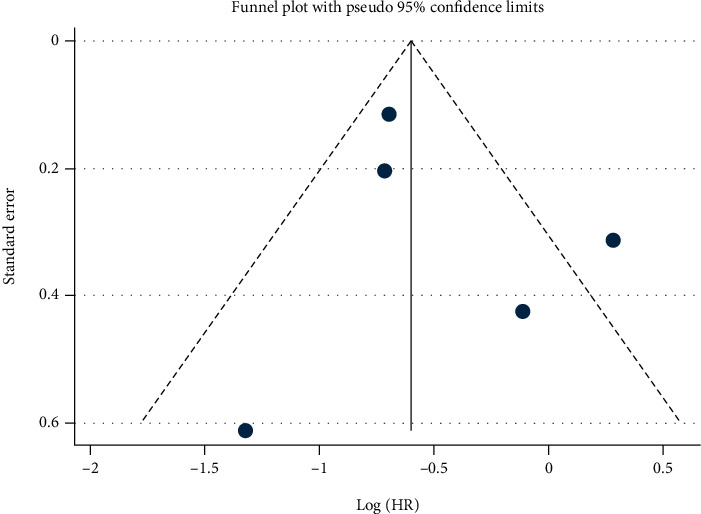
Begg's funnel plot for the prognostic meta-analysis.

Five studies were used to assess the OS shown in Figure 11. There was statistically significant heterogeneity (*I*^2^ = 65.9%, *p* = 0.019), so a random effects model was used. The pooled HR was 0.62 (95%CI = 0.41-0.93, *p* = 0.020), suggesting that a high level of miR-192 was associated with positive patients' survival. The funnel plot was symmetrical, and Begg's test (*p* = 0.806) also indicated that there was no publication bias (Figure 12).

## 4. Discussion

At present, imaging examination is used as a means of preliminary diagnosis and the final diagnosis still relies on the invasive biopsy. Once discovered, most cancers have entered the advanced stage, which causes great difficulties in treatment. Moreover, biopsy progress is invasive and may cause tumor dissemination [31]. Therefore, cancers might be detected at an early stage, if some biomarkers can be used for tumor screening clinically, providing the possibility of a cure. It is also possible to judge the prognosis by biomarkers, which is convenient, fast, and economical.

miRNA is a type of small noncoding RNA that plays important regulatory roles in gene expression and various biological processes [32]. Recently, miRNAs have been shown to have the potential to predict the diagnosis and prognosis of cancer patients [33], which could be used as diagnostic and prognostic biomarkers. miR-192 is one of those miRNAs reported to be a potential diagnostic and prognostic marker [23, 28].

Some studies have revealed that miR-192 was associated with the progression of cancers. miR-192 inhibits cell proliferation, induces apoptosis, and is a positive prognostic factor in human breast cancer [12], acute lymphoblastic leukemia [34], osteosarcoma [11, 35], and colon cancer [9]. However, miR-192 also induces the proliferation and is a poor prognostic factor in neuroblastoma [8], gastric cancer [6], NSCLC [14], cholangiocarcinoma [19], hepatocellular carcinoma [25], pancreatic cancer [26], cervical cancer [15], bladder cancer [16], and AML [23].

The roles of miR-192 in different types of cancers are controversial. In breast cancer, BMP-6 inhibits cell proliferation through upregulating miR-192 [36], and miR-192 inhibits the proliferation and induces the apoptosis in breast cancer through targeting caveolin 1 [12]. In acute lymphoblastic leukemia, overexpression of miR-192 results in cell proliferation arrest and apoptosis increasing in ALL cells through upregulating P53, BAX, and CASP3 [34]. Upregulation of miR-192 suppresses the progress of osteosarcoma through targeting USP1 [35], TCF7 [37], and XIAP [38]. miR-192 also benefits the prognosis of colon cancer patients by regulating SRPX2 [39], Rab-2A [40], RhoA-ROCK-LIMK2 [41], farnesoid X receptor [42], RB1/E2F1 pathway [43], and NOD2 [44]. miR-192 also suppresses the growth of bladder cancer cells via targeting Yin Yang 1 [45]. However, miR-192 serves as a poor prognosis marker in other cancer types including neuroblastoma targeting Dicer1 [8], gastric cancer targeting RAB11-FIP2 [46] and SMG-1 [47], NSCLC targeting the FGFR3/RB1 pathway [48], hepatocellular carcinoma targeting SEMA3A [49], and pancreatic cancer targeting SIP1 [50]. The summary of different interactions of miR-192 with proteins or genes in different cancer types is shown in Table 4. miR-192 has been reported to be induced or inhibited by different agents. Some agents such as nicotine [48], baicalin [51], sinomenine [52], astragaloside IV [13], captopril and spironolactone [53], paclitaxel [54], and doxorubicin [55] can upregulate miR-192, while other agents including metabolites of intestinal microflora [41], curcumin [56, 57], and lactobacillusin [58] can downregulate the expression of miR-192 (Table 5).

This study is the first meta-analysis regarding the diagnostic and prognostic value of miR-192 in various cancers. The results are as follows: AUC 0.82 (95%CI = 0.78-0.85), sensitivity 0.79 (95%CI = 0.75-0.82), and specificity 0.74 (95%CI = 0.64-0.82), demonstrating that miR-192 might be used as a novel biomarker for the detection of cancers. The pooled DOR was 10.50 (95%CI = 5.89-18.73) suggesting that miR-192 is reliably used in cancer diagnosis.

The pooled PLR was 3.03 (95%CI = 2.11-4.34), and NLR was 0.29 (95%CI = 0.23-0.37), meaning that cancer patients had a higher 3.03-fold probability of being miR-192 positive compared to control patients, and the probability of a negative result in patients was 0.29 times that in nonpatients. Fagan's nomogram revealed that when a pretest probability of 50% was specified, the positive posttest probability would increase to 75%, and the negative posttest probability would decrease to 22%. The results suggest that miR-192 is reliable for the detection and diagnosis in NSCLC, CCA, HCC, PDAC, PC, CC, BC, and AML.

The pooled HR was 0.62 (95%CI = 0.41-0.93, *p* = 0.020), suggesting that a high level of miR-192 was associated with positive patients' survival in GC, CoC, HCC, SCLC, and AML.

In the diagnostic meta-analysis, the sensitivity had no heterogeneity (*I*^2^ = 9.63%, 95%CI = 0-61.53%), but there was significant heterogeneity in the specificity (*I*^2^ = 76.64%, 95%CI = 63.03%-90.24%). One reason for the heterogeneity perhaps was that the ethnicity of patients in most studies was Asian, which may result in the bias. Secondly, the cut-off values in studies were different and some of them were not mentioned. Thirdly, different sample types might contribute to the heterogeneity. However, the ROC plane represented a nontypical shoulder arm-like appearance, and Spearman's correlation coefficient was -0.374 (*p* = 0.258), suggesting that there was no threshold effect. Therefore, the threshold effect was not a major cause of heterogeneity.

For the prognostic meta-analysis, there was also heterogeneity (*I*^2^ = 65.9%, *p* = 0.019). However, meta-regression analyses and subgroup analysis cannot be performed because of insufficient study numbers.

The present study has several limitations: firstly, the number of studies available for meta-analysis was small; secondly, the kind of ethnicity was monotonous; thirdly, the values of cut-off in the studies were partial and different; finally, it is difficult to conduct subgroup analysis, such as the influence of gender and tumor stage on the results due to the limited data of original articles.

## 5. Conclusions

In conclusion, this study demonstrates that miR-192 has a moderate diagnostic value to distinguish cancer patients from controls and also can be a promising positive prognostic biomarker in some types of cancer.

## Figures and Tables

**Table 1:** Main characteristics of the eligible studies for diagnostic meta-analysis.

First author	Year	Country	Ethnicity	Cancer type	Sample type	Gender case/control male (female)	Up-/downregulation	Single/multiple miRNAs	Test method	Cut-off	Cases/controls	TP	FP	FN	TN	QUADAS-2
Bagheri	2017	Iran	Asian	NSCLC	Tissue	15 (2)/15 (2)	Down	Multiple	qRT-PCR	0.53	17/17	12	9	5	8	5
Wali	2014	USA	Caucasian	NSCLC	Tissue	19 (18)/26 (13)	Up	Multiple	qRT-PCR	NA	37/39	33	8	4	31	4
Loosen	2019	Germany	Caucasian	CCA	Serum	54 (40)/31 (9)	Up	Multiple	qRT-PCR	NA	94/40	81	4	13	36	6
Silakit	2014	Thailand	Asian	CCA	Serum	32 (19)	Up	Single	qRT-PCR	0.0054	51/30	38	8	13	22	4
Silakit	2015	Thailand	Asian	CCA	Urine	11 (11)/6 (15)	Up	Multiple	qRT-PCR	0.936	22/21	14	7	8	14	4
Zhu	2017	China	Asian	HCC	Serum	39 (11)/27 (23)	Up	Multiple	qRT-PCR	1.1	50/50	41	17	9	34	6
Zhang	2014	China	Asian	PDAC	Serum	NA	Up	Multiple	qRT-PCR	1.15	70/40	53	18	17	22	5
Zou	2019	China	Asian	PC	Serum	NA	Up	Multiple	qRT-PCR	2	93/71	72	31	21	40	5
Farzanehpour	2019	Iran	Asian	CC	Tissue	NA	Up	Multiple	qRT-PCR	NA	18/36	14	2	5	34	5
Jiang	2020	China	Asian	BC	Urine	82 (36)/79 (41)	Down	Single	qRT-PCR	NA	118/120	91	26	27	94	5
Tian	2018	China	Asian	AML	Serum	57 (40)	Down	Single	qRT-PCR	NA	97/50	77	9	20	41	5

NSCLC: non-small-cell lung cancer; CCA: cholangiocarcinoma; HCC: hepatocellular carcinoma; PDAC: pancreatic ductal adenocarcinoma; PC: pancreatic cancer; CC: cervical cancer; BC: bladder cancer; AML: acute myeloid leukemia; NA: not available; TP: true positive; FP: false positive; FN: false negative; TN: true negative; QUADAS-2: Quality Assessment of Diagnostic Accuracy Studies 2.

**Table 2:** Main characteristics of the eligible studies for prognostic meta-analysis.

Study	Year	Country	Cancer type	Sample type	Gender high/low male (female)	Up-/downregulation	Single/multiple miRNAs	Methods	Cases high/low	Cut-off	Survival	HR (95% CI)
Chen 2014	2014	China	GC	Plasma	NA	Up	Multiple	qRT-PCR	29/33	NA	OS	0.894 (0.391-2.041)
Li 2018	2018	China	CoC	Tissue	20 (18)/20 (40)	Down	Single	qRT-PCR	38/60	97.43	OS	0.500 (0.390-0.610)
Lian 2016	2016	China	HCC	Tissue	NA	Down	Single	qRT-PCR	24/71	NA	OS	0.267 (0.081-0.887)
Mancuso 2016	2016	Italy	SCLC	Tissue	32 (18)	Up	Multiple	qRT-PCR	25/25	13.59	OS	1.330 (0.720-2.450)
Tian 2018	2018	China	AML	Serum	31 (19)/26 (21)	Down	Single	qRT-PCR	50/47	NA	OS	0.490 (0.342-0.758)

GC: gastric cancer; CoC: colon cancer; SCLC: small-cell lung cancer; HCC: hepatocellular carcinoma; AML: acute myeloid leukemia; high: high miR-192 expression; low: low miR-192 expression; NA: not available; HR: hazard ratio.

**Table 3:** Newcastle–Ottawa Scale scores of studies.

Study	Selection	Comparability	Outcome	Total
Chen 2014	★★★★		★★	6
Li 2018	★★★★	★	★★	7
Lian 2016	★★★★	★		5
Mancuso 2016	★★★★		★★	6
Tian 2018	★★★★	★	★★	7

**Table 4:** Proteins interacting with miR-192 in different cancer types.

Prognosis	Cancer type	miR-192-related regulatory proteins	References
Positive	Breast cancer	BMP-6/miR-192/caveolin 1	[12, 36]
	ALL	miR-192/P53, BAX, CASP3	[34]
	OS	miR-192/USP1, TCF7, XIAP	[35, 37, 38]
	Colon cancer	miR-192/SRPX2, Rab-2A, RhoA-ROCK-LIMK2, farnesoid X receptor, RB1-E2F1, NOD2	[39–44]
	HCC	miR-192/SLC39A6-SNAIL	[29]
	BC	miR-192/transcription factor Yin Yang 1	[45]

Negative	NB	miR-192/Dicer1	[8]
	GC	miR-192/RAB11-FIP2, SMG-1	[46, 47]
	NSCLC	miR-192/FGFR3-RB1	[48]
	PDAC	miR-192/SIP1	[50]

ALL: acute lymphoblastic leukemia; OS: osteosarcoma; HCC: hepatocellular carcinoma; NB: neuroblastoma; BC: bladder cancer; GC: gastric cancer; NSCLC: non-small-cell lung carcinoma; PDAC: pancreatic ductal adenocarcinoma.

**Table 5:** Agents that induce or inhibit miR-192.

Up-/downregulation of miR-192	Agents	Reference
Down	Nicotine	[48]
	Baicalin	[51]
	Sinomenine	[52]
	Astragaloside IV	[13]
	Captopril and spironolactone	[53]
	Paclitaxel	[54]
	Doxorubicin	[55]

Up	Metabolites of intestinal microflora	[41]
	Curcumin	[56, 57]
	Lactobacillusin	[58]

## Data Availability

All data generated or analyzed during this study are included in this article. More information concerning the data can be obtained from the corresponding author.
